# The effect of blood flow restriction combined with different resistance training on the improvement of athletic performance in male tactical personnel

**DOI:** 10.3389/fphys.2026.1855088

**Published:** 2026-06-08

**Authors:** Chunwei Hou, Zichao Zhang, Yao Zhao, Kaidong Liu, Wenwen Liang, Chunhui Wang, Nan Jia, Xintong Li

**Affiliations:** 1 Army Engineering University of People’s Liberation Army (PLA), Shijiazhuang, China; 2 Rehabilitation Medical College, Shandong Second Medical University, Weifang, China; 3 Rehabilitation Medical College, Shandong University of Traditional Chinese Medicine, Jinan, China; 4 School of Humanities, Xi’an Conservatory of Music, Xi’an, China; 5 School of Physical Education &Health (Public Sport Department), Nanning Normal University, Nanning, China; 6 Department of Rehabilitation Medicine, Qingdao Municipal Hospital, Qingdao, China; 7 The Key Laboratory of Biomedical Information Engineering of Ministry of Education, Institute of Health and Rehabilitation Science, School of Life Science and Technology, Xi’an Jiaotong University, Xi’an, China

**Keywords:** athletic performance, blood flow restriction, different load, resistance training, tactical personnel

## Abstract

**Purpose:**

This study aimed to compare the effects of resistance training programs combining different load intensities with blood flow restriction (BFR), alongside a traditional high-load resistance training condition, on athletic performance in male tactical personnel.

**Methods:**

Sixty healthy male tactical personnel with systematic resistance training experience were randomly assigned to four groups (n=15 each): HL-BFR group (70% one repetition maximum [1RM] with BFR), ML-BFR group (50% 1RM with BFR), LL-BFR group (30% 1RM with BFR), and HL-CON group (70% 1RM without BFR), except for load intensity and BFR application. Notably, non-BFR control conditions were only included for the 70% 1RM group. All groups received an 8-week intervention with 3 training sessions per week, and consistent training variables except for load intensity and BFR application. Before and after the 8-week intervention, assessments were conducted for muscle strength (1RM of biceps curl, bench press, deadlift, squat), explosive power (countermovement jump, CMJ), athletic performance (pull-ups, biceps curl, zigzag run, standing long jump, medicine ball throw, 30-meter sprint), body composition (body mass, body fat percentage, lean mass), and serum hormonal levels (testosterone, cortisol, growth hormone [GH]). Heart rate and rating of perceived exertion (RPE) were monitored during training to evaluate training load.

**Results:**

Significant group × time interactions were observed for pull-ups, parallel bar dips, standing long jump, medicine ball throw, and 30-m sprint performance (all p < 0.05). Overall, the HL-BFR and ML-BFR groups demonstrated greater improvements than the LL-BFR group across several strength- and power-related outcomes, whereas differences between HL-BFR, ML-BFR, and HL-CON were generally small and inconsistent. CMJ performance improved over time in all groups without significant between-group differences. No significant interaction effect was identified for zigzag run performance. Resting hormonal concentrations and body mass remained unchanged across groups (all p > 0.05). Small increases in segmental lean mass were observed in the HL-BFR and ML-BFR groups; however, these changes should be interpreted cautiously due to the limited magnitude of change and the measurement variability associated with bioelectrical impedance analysis. RPE and heart rate decreased over time during the intervention period (all p < 0.001), with no consistent between-group differences.

**Conclusions:**

Under the specific training conditions employed in this study, resistance training combined with ML−BFR and HL−BFR generally produced greater improvements in athletic performance outcomes than LL−BFR. However, high-load BFR did not consistently outperform traditional high-load resistance training without BFR. These findings should therefore be interpreted as differences among specific load–BFR combinations rather than the isolated effect of BFR itself. Moderate-load BFR may represent a practical strategy for improving performance while reducing mechanical loading demands, although caution is warranted when generalizing beyond the present protocol.

## Introduction

Blood flow restriction (BFR) training involves the application of external pressure to the proximal limb to maintain arterial inflow while intermittently restricting venous return during exercise (Loenneke and Pujol, 2009), thereby augmenting metabolic stress, promoting muscle protein synthesis, and enhancing muscle hypertrophy and mass (Reeves et al., 2006). In recent years, BFR has been proven to effectively promote muscle hypertrophy and strength improvement in healthy individuals (Yasuda et al., 2011), the elderly (Karabulut et al., 2011), and those undergoing rehabilitation (Ohta et al., 2003), and has been widely applied in competitive sports (Takarada et al., 2004).

BFR training at different load levels may produce different adaptive effects, but the specific differences are not yet clear. Low-load BFR (20%–30% one repetition maximum [1RM]) can induce comparable muscular adaptations to high-load resistance training (HL-RT) (Yasuda et al., 2011; Laurentino et al., 2012). However, some studies have indicated that HL-RT(70%–85% 1RM) may be more advantageous in terms of strength improvement (Yasuda et al., 2011; Martín-Hernández et al., 2013; Vechin et al., 2015), suggesting that relying solely on metabolic stress may not be able to completely replace the stimulation of high mechanical tension. Meanwhile, the high-load BFR training (HL-BFRT) remains a subject of controversy. Cook et al. (2014) reported that it can further enhance strength and power output, while others (Laurentino et al., 2008) have not observed any additional benefits compared to HL-RT. This inconsistency may stem from differences in load selection, pressure application methods, training context, and movement patterns (Abe et al., 2005; Laurentino et al., 2008; Cook et al., 2014). This issue is particularly critical in tactical personnel. Tactical personnel need to possess high levels of strength, explosive power, and fatigue resistance simultaneously during high-intensity training and complex tactical missions. While HL-RT can effectively improve specialized military performance (Harman et al., 1997; Knapik, 1997), its significant mechanical stress may increase the risk of fatigue accumulation and sports injuries. On the other hand, while low-load training is relatively safe, it may not provide sufficient mechanical stimulation to meet the demands of actual combat. Therefore, exploring effective training strategies to further enhance athletic performance while ensuring training safety has become a crucial issue that urgently needs to be addressed in current tactical personnel training. Based on this, this study systematically compares the effects of BFR under different load conditions and HL-RT on muscle strength, explosive power, and related physiological indicators, taking tactical personnel as the subjects. The aim is to clarify the adaptation characteristics and application value of BFR under different loads, and to provide a scientific basis for optimizing tactical training programs.

## Methods

### Experimental design

This study employed a randomized, four-arm, parallel-group, single-blind controlled trial design, in which outcome assessors and data analysts were blinded to group allocation. The study was approved by the Ethics Committee of Nanning Normal University and was conducted in accordance with the Declaration of Helsinki (LLSC2025022). Informed consent was obtained from all participants before participating in the experiment.

### Participants

**Figure 1:**
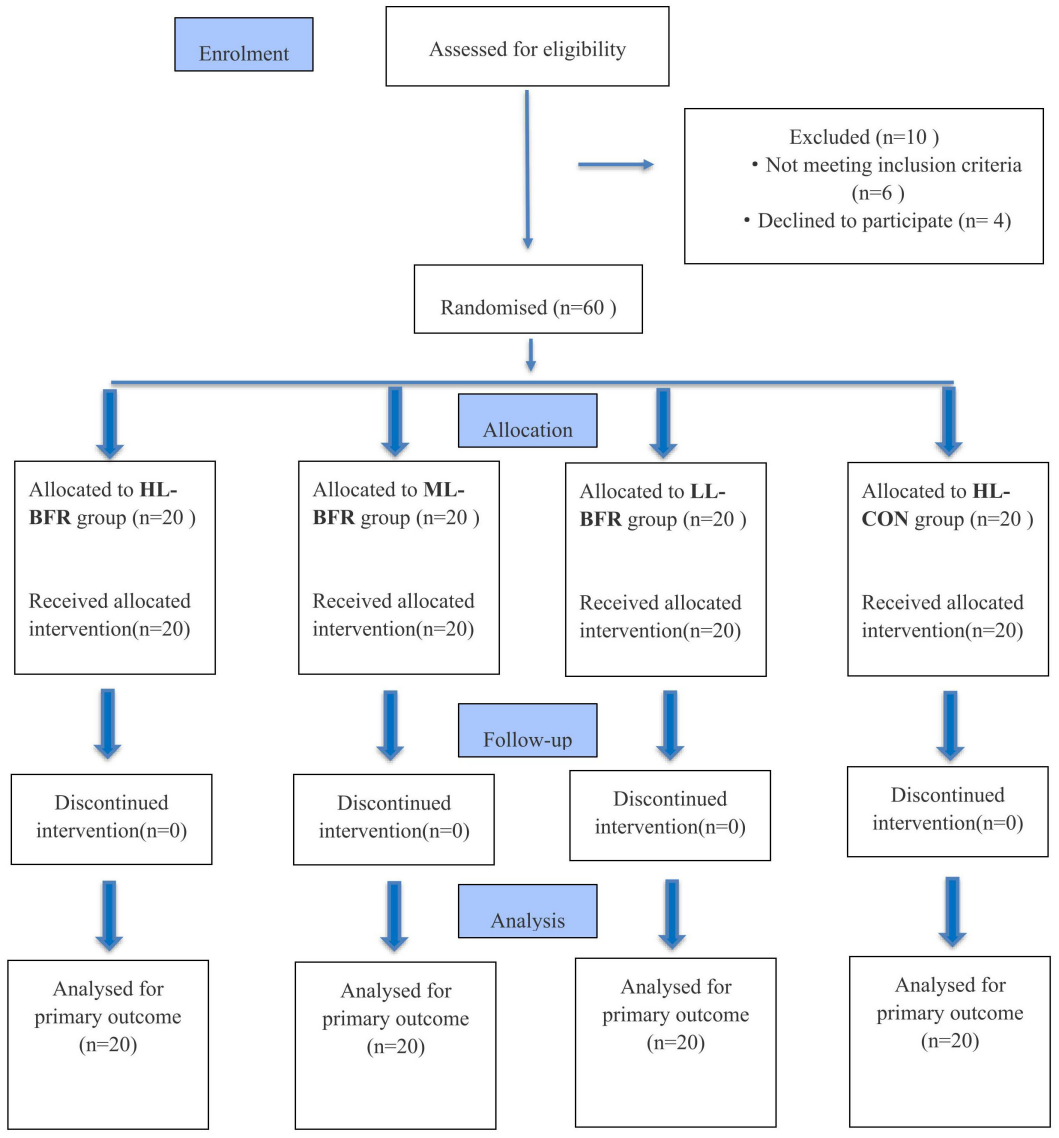
CONSORT flow diagram.

A prospective power analysis was conducted using G*Power (version 3.1.9.7) to determine the required sample size for a repeated measures ANOVA (within–between interaction). Based on previous research (Harman et al., 2008), an effect size of f = 0.2526 (derived from the calculated value), α = 0.05, power (1–β) = 0.90, four groups, and two measurement time points, the required total sample size was calculated to be 44 participants. The assumed correlation among repeated measures was set at 0.5, with a nonsphericity correction ϵ of 1. A total of 60 participants were ultimately enrolled in the present study. The assumed correlation among repeated measures was set at 0.5, with a nonsphericity correction ϵ of 1. Inclusion criteria were: (a) healthy males aged 18–25 years with at least 6 months of systematic resistance training experience, (b) familiarity with basic strength training movements and (c) no BFR training in the 3 months prior to the start of the study; no history of cardiovascular, metabolic or neurological diseases, and no musculoskeletal injuries affecting normal training in the past 6 months. Exclusion criteria included: (a) presence of contraindications to BFR training (such as history of thrombosis, hypertension or peripheral vascular disease, etc.), (b) recent use of drugs or supplements that may affect muscle adaptation or hormone levels and (c) injuries or diseases that affect training or testing during the study. All participants provided written informed consent for participation. The flow of participants through the study is summarized in **Figure 1**. A total of 60 participants were screened, and 60 were ultimately enrolled and completed.

### Experimental procedure

Standardized assessments were performed before and after the 8-week intervention period. All participants completed a battery of tests evaluating hormonal responses, body composition, explosive performance, and athletic performance.

### Pre test

#### Day1

##### Hormones

Blood samples (5 ml) were collected from the antecubital vein and placed in EDTA tubes. Immediately after collection, samples were centrifuged (4 °C, 3000 rpm) for 10 minutes to separate the serum, and then stored at -20 °C until subsequent batch analysis. Free testosterone (Testosterone Kit IBL, RE52151, Hamburg, Germany), cortisol (Cortisol Kit IBL, RE52061, Hamburg, Germany), and growth hormone (GH) (Growth Hormone Kit IBL, MG59121, Hamburg, Germany) in the serum samples were analyzed by enzyme immunoassay. The inter- and intra-assay coefficients of variation for hormone measurements were 5.5% and 3.1% (testosterone), 3.4% and 2.6% (cortisol), and 8.1% and 4.9% (GH), respectively.

##### Body composition

Body composition indices, including BMI, were measured using a bioelectrical impedance analysis system (TANITA BC-720, Tanita Corporation, Tokyo, Japan). Assessments were conducted in the morning following an overnight fast, with participants instructed to empty their bladders, and avoid strenuous exercise prior to testing. The device was calibrated before each session. Participants, wearing minimal clothing and no metal objects, stood upright on the analyzer with arms extended at approximately 45° to the body, remaining still until completion.

##### CMJ height

The CMJ was performed on an infrared platform (Optojump, Microgate), which calculated jump height based on flight time and gravitational acceleration (h = t^2^ × g/8).Participants performed the jump with both hands placed on the waist, starting from an upright position, followed by a downward movement to approximately 90° of knee flexion and an immediate maximal vertical jump. Each subject completed three trials with 1-minute rest intervals, and the mean jump height was recorded.

##### Maximum strength test

Prior to 1RM testing, participants performed a standardized warm-up consisting of 1–5 submaximal sets at 40%–80% of the estimated 1RM, with 1–10 repetitions per set. A brief low-intensity aerobic warm-up was also included. The test exercises included bicep curls, bench press, deadlift, and squat, and the 1RM for each exercise was assessed separately. The 1RM was determined using a progressive loading protocol, with loads increased incrementally until failure. Participants completed 3–8 attempts, with the load increased after a successful lift and reduced or terminated after a failed attempt. Rest intervals between attempts ranged from 2 to 5 minutes to ensure adequate recovery. The 1RM was defined as the maximum load lifted once with proper technique through the full range of motion without assistance (Grgic et al., 2020).

#### Day 2

##### Athletic performance

Twenty-four hours after day 1 testing, performance assessments were conducted, including pull-ups, biceps curl, zigzag run, standing long jump, medicine ball throw, and 30-meter sprint. Pull-ups and biceps curl repetitions were manually counted and recorded by the same trained assessor. Zigzag run and 30-meter sprint times were measured using a handheld stopwatch (or electronic timing system, if applicable) to the nearest 0.01 s. Standing long jump and medicine ball throw distances were measured using a standardized measuring tape to the nearest 0.1 cm. All assessments were performed indoors under standardized environmental conditions.

Pull-ups: Subjects complete as many standard pull-ups as possible within 2 minutes, ensuring the chin is above the bar and arms are fully extended. The number of repetitions is counted. Parallel bar dips: Subjects complete as many triceps extensions as possible on parallel bars within 2 minutes, with elbows bent at approximately 90° during descent. The number of repetitions is counted. Zigzag run: Subjects complete a 25-meter serpentine run around five cones spaced 5 meters apart at their fastest speed. The completion time (in seconds) is recorded. Standing long jump: Subjects stand with feet together behind the take-off line and jump forward. The farthest distance (in centimeters) from three attempts is recorded as the score. Medicine ball throw: From a standing position, participants performed a maximal two-handed vertical throw with a 4-kg medicine ball. The farthest distance (m) from three attempts is recorded. 30-meter sprint: Subjects started from a standing position 0.5 m behind the line, sprinting maximally through the finish. The fastest of three trials was recorded, with 2-min rests between attempts. Assessments are conducted indoors to prevent environmental influences.

##### Experimental intervention

After baseline testing, participants were randomly assigned to four groups (n = 15 per group) using a computer-generated random sequence. The four groups were: HL-BFR group (70% 1RM with BFR), ML-BFR group (50% 1RM with BFR), LL-BFR group (30% 1RM with BFR), and HL-CON group (70% 1RM without BFR). Based on the established 1RM, training loads at 30%, 50%, and 70% were calculated by multiplying individual 1RM values by 0.3, 0.5, and 0.7, respectively, and rounding to the nearest load increment permitted by the equipment. Microloading was applied when necessary. The calculated loads were verified during a familiarization session, with minor adjustments made if perceived exertion did not match the intended intensity. Loads were re-evaluated periodically every 1–2 weeks to maintain consistent relative intensity throughout the intervention. The training intervention lasted for 8 weeks and was performed three times per week, with all sessions conducted in the morning. Detailed training protocols are presented in **Table 1**. All exercises were performed for 4 sets of 8 repetitions with controlled tempo and full range of motion under supervision. Sets were terminated if improper technique, pain, or loss of control occurred. For BFR conditions, pneumatic cuffs were applied bilaterally to the proximal limbs, with pressure set at 50% of individual limb occlusion pressure (LOP) (Patterson et al., 2019), determined prior to training, and maintained during each exercise bout. The cuffs remained inflated during each exercise bout, including inter-set rest intervals, and were deflated between exercises. Rest intervals were 30–60 s, and total occlusion time per exercise did not exceed 10–15 min. Participants were monitored for adverse symptoms, and no adverse events were reported. Participants followed dietary control and abstained from alcohol throughout the intervention. Heart rate and rating of perceived exertion (RPE) were recorded at weeks 1, 4, and 8. The 1RM was reassessed after week 4 to adjust training loads.

**Table 1:** Exercise prescription for all groups.

Days	Exercise	Sets	Repetitions	Rest
Day1	Bent-over row	4	8	2min
	Bent-over reverse fly	4	8	2min
	Barbell bench press	4	8	3min
	Hang power clean	4	8	3min
Day3	Back squat	4	8	2min
	Deadlift	4	8	2min
	Hip thrust	4	8	3min
	Box squat	4	8	3min
Day5	Weighted pull-up	4	8	2min
	Weighted dip	4	8	2min
	Incline dumbbell press	4	8	3min
	Hang power clean	4	8	3min

##### BFR

Before training, LOP was measured using Doppler ultrasound (TE7, Mindray, China) with a pneumatic cuff (Theratools, China; 68 cm length, 7.5 cm width) placed at the proximal arm or thigh. For the upper limbs (Pişkin et al., 2024), pressure was increased from 50 mmHg in 1-mmHg increments until radial artery pulse disappearance. For the lower limbs (Mattocks et al., 2017), following inflation to 50 mmHg for 30 s and 10 s deflation, pressure was increased in 10-mmHg increments until posterior tibial artery pulse cessation. The lowest pressure without an audible pulse was defined as LOP.

##### Heart rate and RPE

Heart rate was continuously monitored via a telemetry system (Polar RC3, Polar Electro Oy) at weeks 1, 4, and 8, and RPE was assessed immediately after the completion of each exercise protocol using the Borg 6–20 Rating of Perceived Exertion scale.

### Post assessments

**Figure 2:**
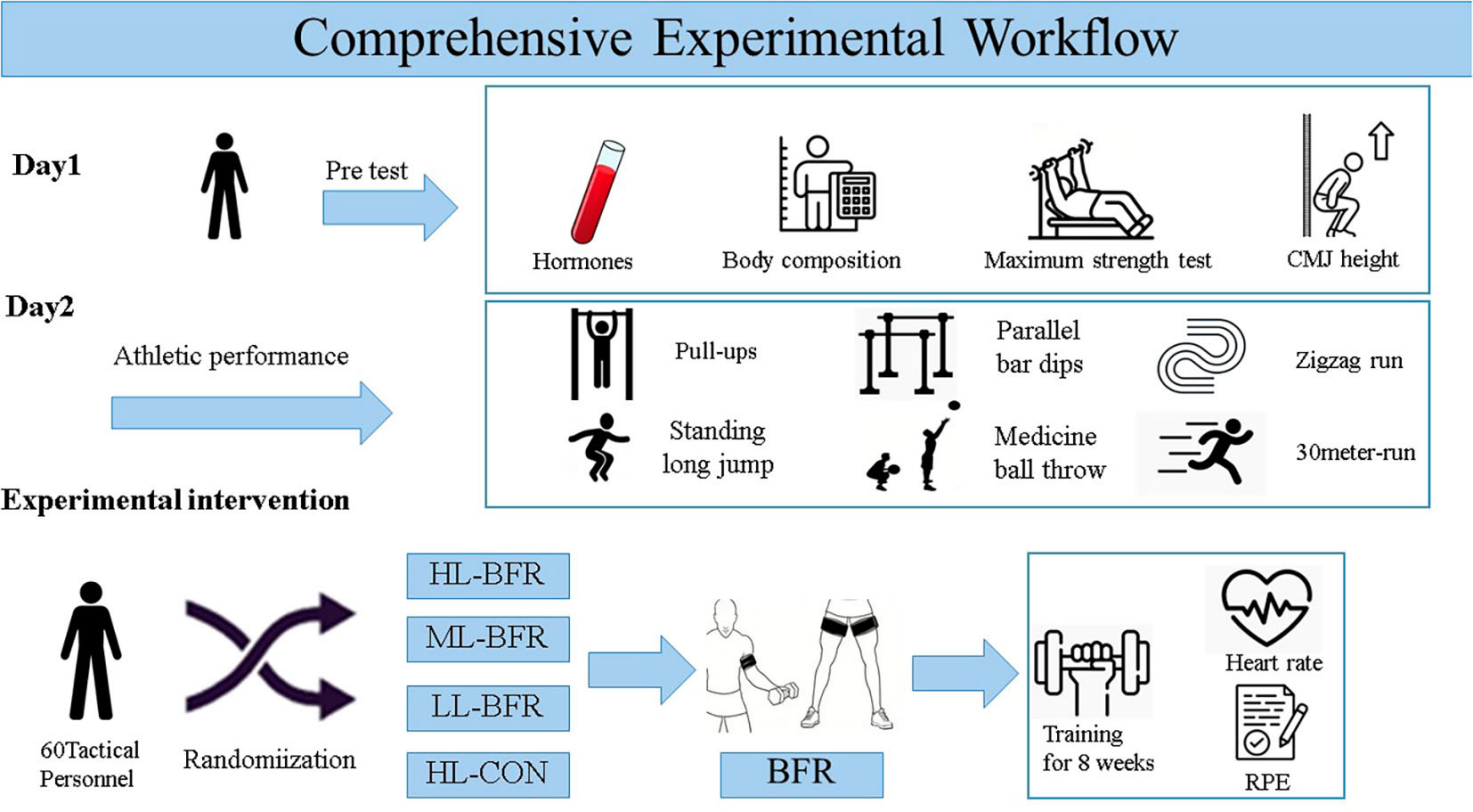
The test process. RPE, rating of perceived exertion.

The pre-assessment process is repeated 48 hours after the last intervention and the entire testing process is shown in **Figure 2**.

### Statistical analysis

All statistical analyses were performed using IBM SPSS Statistics Version 27.0 (IBM Corp., Armonk, NY, USA). Data normality was assessed using the Shapiro–Wilk test. Normally distributed variables were expressed as mean ± standard deviation (SD).Baseline differences among groups were evaluated using one-way analysis of variance (ANOVA) for normally distributed variables and the Kruskal–Wallis H test for non-normally distributed variables.

For normally distributed outcomes, a 4 (group) × 2 (time) mixed-design repeated-measures analysis of variance (rmANOVA) was conducted to examine the main effects of group and time, as well as the group × time interaction effect. Mauchly’s test was used to assess sphericity, and Greenhouse–Geisser corrections were applied when the assumption of sphericity was violated.

When significant interaction or main effects were observed, Bonferroni-adjusted *post hoc* comparisons were performed. For non-normally distributed variables, analysis of covariance (ANCOVA) was applied to compare post-intervention values among groups while adjusting for baseline values.

Heart rate and rating of perceived exertion (RPE) measured repeatedly during the intervention were analyzed using generalized estimating equations (GEE), including group, time, and group × time interaction terms to account for within-subject correlations.

Effect sizes for rmANOVA were reported as partial eta-squared (η_p_^2^), with thresholds of 0.01, 0.06, and 0.14 representing small, medium, and large effects (Hopkins et al., 2009), respectively.

## Results

### Participants

A total of 60 participants were included and randomly assigned to four groups. Baseline demographic and anthropometric characteristics of the participants are presented in **Table 2**. No significant between-group differences were observed at baseline for age, height, body mass, or body fat percentage (all p > 0.05), indicating that the groups were comparable prior to the intervention.

**Table 2:** Baseline anthropometric characteristics.

Measure	HL-BFR	ML-BFR	LL-BFR	HL-CON	*F*	*P*
Age(y)	24.40 ± 1.10	23.90 ± 1.40	23.70 ± 1.30	23.30 ± 1.10	2.12	0.100
Height(cm)	177.00 ± 3.90	174.80 ± 6.40	173.60 ± 4.40	175.00 ± 4.4	1.25	0.300
Body mass(kg)	71.70 ± 7.10	71.20 ± 11.50	71.20 ± 8.70	70.60 ± 8.60	0.04	0.990
Body fat(%)	18.00 ± 6.10	16.40 ± 4.70	18.80 ± 4.90	18.50 ± 5.60	0.58	0.630

Values are presented as mean ± SD, HL-BFR, 70%1RM with BFR; ML-BFR, 50%1RM with BFR; LL-BFR, 30%1RM with BFR; HL-CON, 70%1RM without BFR.

### Athletic performance

**Figure 3:**
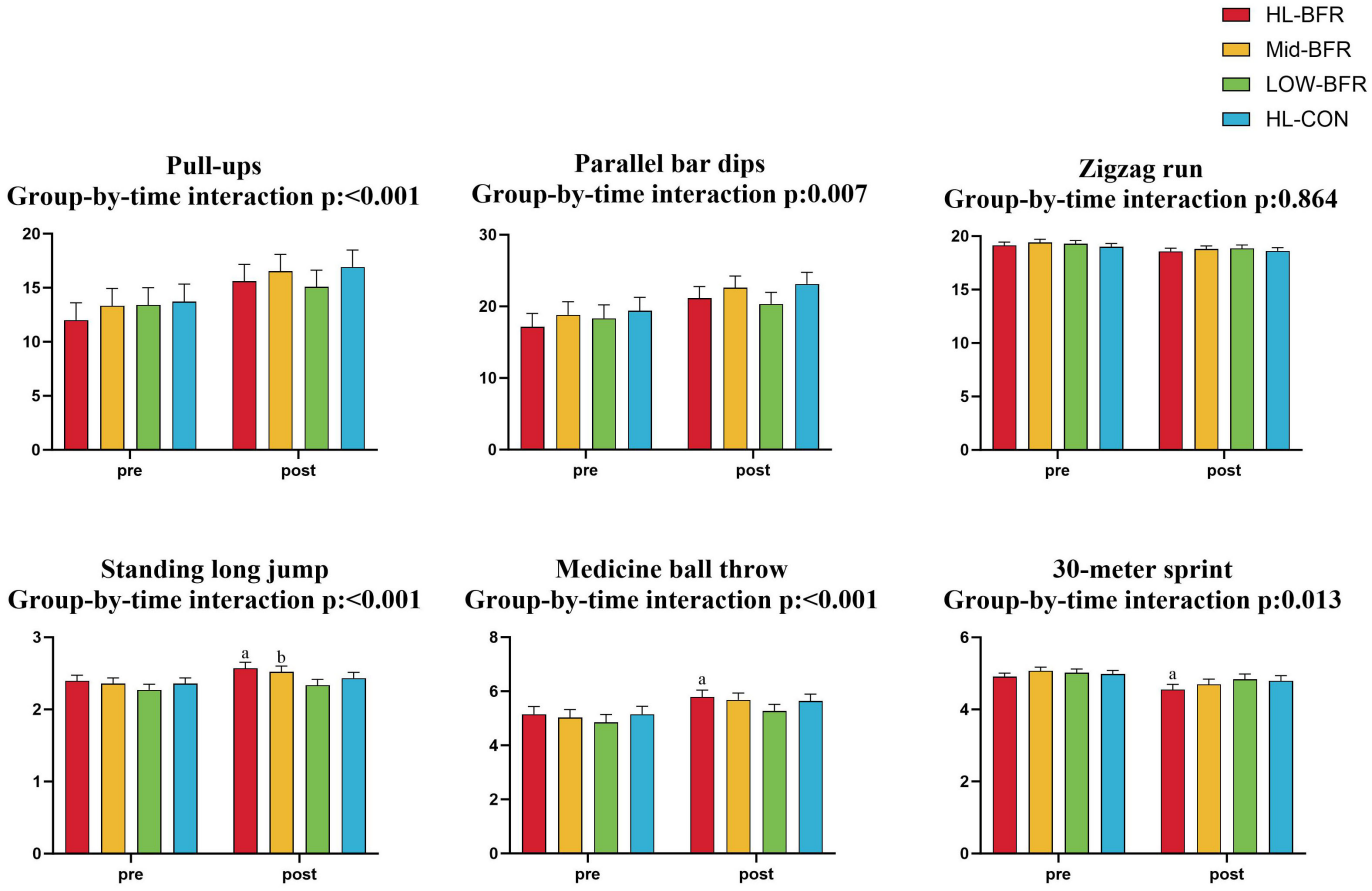
Performance levels before and after training. Values are presented as mean ± S. HL-BFR, 70%1RM with BFR; ML-BFR, 50%1RM with BFR; LL-BFR, 30%1RM with BFR; HL-CON, 70%1RM without BFR. (a) indicates a significant between-group difference at post-intervention between the HL-BFR and LL-BFR groups (p < 0.05). (b) indicates a significant between-group difference at post-intervention between the ML-BFR and LL-BFR groups (p < 0.05).

**Figure 3** shows the changes in motor performance before and after intervention in each group. No significant between-group differences were observed in motor performance at baseline (all p > 0.05).

### Pull-ups

A significant group-by-time interaction was observed for pull-up performance (P < 0.001). *Post-hoc* analysis revealed that all four groups exhibited significant improvements from pre- to post-intervention (all within-group P < 0.001). The HL-BFR group demonstrated the largest increase (Δ = 3.60 repetitions, 95% CI: 3.26 to 3.94, ηp^2^ = 0.891), followed by the ML-BFR group (Δ = 3.20, 95% CI: 2.86 to 3.54, ηp^2^ = 0.866) and the HL-CON group (Δ = 3.20, 95% CI: 2.86 to 3.54, ηp^2^ = 0.866), while the LL-BFR group showed the smallest improvement (Δ = 1.67, 95% CI: 1.33 to 2.00, ηp^2^ = 0.637). These findings suggest that medium- and high-load BFR protocols may provide a more effective training stimulus than low-load BFR for improving pull-up performance.

### Parallel bar dips

A significant group-by-time interaction effect was detected for parallel bar dips (P = 0.007). All groups showed significant improvements across time points (all within-group P < 0.001). The HL-BFR group exhibited the greatest improvement (Δ = 4.00 repetitions, 95% CI: 3.12 to 4.88, ηp^2^ = 0.598), followed by the ML-BFR group (Δ = 3.80, 95% CI: 2.92 to 4.68, ηp^2^ = 0.573) and the HL-CON group (Δ = 3.73, 95% CI: 2.86 to 4.61, ηp^2^ = 0.564). The LL-BFR group showed the smallest increase (Δ = 2.00, 95% CI: 1.12 to 2.88, ηp^2^ = 0.271). These results suggest that both HL-BFR and ML-BFR are effective in improving upper-body pushing performance, with high-load conditions yielding marginally larger gains.

### Zigzag run

No significant group-by-time interaction was found for zigzag run performance (P = 0.864), indicating that the rate of change did not differ across groups. A significant main effect of time was observed (P < 0.001), reflecting overall improvement in agility performance in all groups. The ML-BFR group showed the greatest reduction in completion time (Δ = −0.61 s, 95% CI: −1.06 to −0.17, ηp^2^ = 0.120), followed by the HL-BFR group (Δ = −0.57 s, 95% CI: −1.01 to −0.11, ηp^2^ = 0.103), the LL-BFR group (Δ = −0.43 s, 95% CI: −0.87 to 0.01, ηp^2^ = 0.063), and the HL-CON group (Δ = −0.38 s, 95% CI: −0.82 to 0.06, ηp^2^ = 0.050). The lack of interaction suggests that BFR training, regardless of load intensity, does not differentially affect agility compared to conventional training.

### Standing long jump

A significant group-by-time interaction was identified for standing long jump performance (P < 0.001). The time effect was also significant (P < 0.001). The HL-BFR group demonstrated the largest improvement (Δ = 0.17 m, 95% CI: 0.13 to 0.21, ηp^2^ = 0.568), closely followed by the ML-BFR group (Δ = 0.16 m, 95% CI: 0.12 to 0.20, ηp^2^ = 0.523). In contrast, the LL-BFR group (Δ = 0.06 m, 95% CI: 0.02 to 0.10, ηp^2^ = 0.154) and HL-CON group (Δ = 0.07 m, 95% CI: 0.03 to 0.11, ηp^2^ = 0.197) showed substantially smaller gains. These findings indicate that higher-load BFR protocols (HL-BFR and ML-BFR) are superior to low-load BFR and conventional training for improving lower-body explosive power.

### Medicine ball throw

A significant group-by-time interaction was observed for medicine ball throw performance (P < 0.001), along with a significant main effect of time (P < 0.001). The HL-BFR group demonstrated the greatest increase (Δ = 0.17 m, 95% CI: 0.13 to 0.21, ηp^2^ = 0.568), followed by the ML-BFR group (Δ = 0.16 m, 95% CI: 0.12 to 0.20, ηp^2^ = 0.523). The LL-BFR group (Δ = 0.06 m, 95% CI: 0.02 to 0.10, ηp^2^ = 0.154) and HL-CON group (Δ = 0.07 m, 95% CI: 0.03 to 0.11, ηp^2^ = 0.197) exhibited more modest improvements. The pattern of results mirrors that of the standing long jump, reinforcing that combined high-load and BFR stimuli produce the most pronounced gains in upper-body explosive power.

### 30-meter sprint

A significant group-by-time interaction was found for 30-meter sprint performance (P = 0.013). The main effect of time was also significant (P < 0.001). The ML-BFR group showed the greatest reduction in sprint time (Δ = −0.37 s, 95% CI: −0.48 to −0.27, ηp^2^ = 0.481), followed closely by the HL-BFR group (Δ = −0.35 s, 95% CI: −0.46 to −0.25, ηp^2^ = 0.452). The HL-CON group (Δ = −0.19 s, 95% CI: −0.29 to −0.08, ηp^2^ = 0.192) and LL-BFR group (Δ = −0.18 s, 95% CI: −0.28 to −0.07, ηp^2^ = 0.181) demonstrated smaller improvements. These results suggest that moderate- and high-load BFR training are more effective than low-load BFR and conventional training in improving sprint performance.

### Hormones

Baseline hormone levels were comparable across groups (all p > 0.05). No significant pre–post changes were observed in resting growth hormone, cortisol, or testosterone levels in any group (all p > 0.05). Between-group comparisons also revealed no significant differences in hormonal changes. It should be noted that only chronic resting hormone concentrations were assessed in the present study, and acute post-exercise hormonal responses were not evaluated. Some moderate effect sizes were observed, but these did not reach statistical significance and therefore require cautious interpretation. Experimental data are shown in **Table 3**.

**Table 3:** Serum hormone levels.

Variables	Group	Pre	Post	Δ Change (95%CI)	Within-group effect size (Pη^2^)	Time effect *p*	Group-by-time interaction *p*
GH(ng/ml)	HL-BFR	0.84 ± 0.08	0.87 ± 0.11	0.03(-0.01,0.07)	0.044	0.005	0.473
	ML-BFR	0.82 ± 0.06	0.88 ± 0.10	0.03(-0.01,0.06)	0.037		
	LL-BFR	0.87 ± 0.09	0.87 ± 0.12	0.01(-0.03,0.04)	0.001		
	HL-CON	0.83 ± 0.11	0.88 ± 0.03	0.05(0.01,0.08)	0.102		
Cortisol(ng/ml)	HL-BFR	104.25 ± 5.84	106.83 ± 7.61	0.58(-0.01,1.16)	0.065	0.001	0.984
	ML-BFR	102.13 ± 4.29	104.32 ± 6.51	0.42(-0.16,1.00)	0.036		
	LL-BFR	102.89 ± 7.71	103.71 ± 5.63	0.53(-0.05,1.11)	0.056		
	HL-CON	107.28 ± 7.96	109.13 ± 6.01	0.48(-0.10,1.06)	0.046		
Testosterone(ng/ml)	HL-BFR	5.52 ± 0.68	6.10 ± 0.88	2.59(-0.04,5.21)	0.065	0.129	0.121
	ML-BFR	5.13 ± 0.73	5.55 ± 0.89	2.20(-0.43,4.83)	0.048		
	LL-BFR	4.98 ± 0.74	5.51 ± 1.00	0.87(-1.81,3.45)	0.007		
	HL-CON	5.32 ± 0.80	5.80 ± 0.91	-1.55(-4.18,1.08)	0.024		

Values are presented as mean ± SD. HL-BFR, 70%1RM with BFR; ML-BFR, 50%1RM with BFR; LL-BFR, 30%1RM with BFR; HL-CON, 70%1RM without BFR.

### Body composition

For body composition, small but statistically significant increases were observed in the left arm and left leg of the HL-BFR and ML-BFR groups (both p < 0.05), whereas no significant changes were found in the LL-BFR or HL-CON groups. However, the absolute magnitude of these changes was minimal and should be interpreted cautiously given the inherent measurement variability associated with bioelectrical impedance analysis. No consistent between-group differences were identified for lean mass in other limb segments, and no significant interaction effect was observed for any lean mass variable. Specific data are presented in **Table 4**.

**Table 4:** Strength levels before and after training.

Variables	Group	Pre	Post and adjusted post	Δ Change (95%CI)	Within-group or group effect size(pη^2^)	Time effect p or ANCOVA *p*	Group-by-time interaction *p*
CMJ height(cm)	HL-BFR	50.45 ± 5.73	53.74 ± 5.81	3.29(2.67,3.90)	0.672	<0.001	0.064
	ML-BFR	51.36 ± 5.70	54.55 ± 4.70	3.19(2.57,3.80)	0.658		
	LL-BFR	49.63 ± 6.46	51.91 ± 5.56	2.29(1.67,2.90)	0.498		
	HL-CON	50.49 ± 6.46	53.02 ± 6.89	2.53(1.91,3.14)	0.548		
Bicep curls(kg)	HL-BFR	14.33 ± 1.18	17.70 ± 1.36 19.12 ± 0.34a	3.37(2.74,4.00)	0.369	<0.001	/
	ML-BFR	14.83 ± 0.49	17.67 ± 1.23 18.63 ± 0.33b	2.83(2.21,3.46)			
	LL-BFR	17.00 ± 0.06	17.97 ± 1.30 16.96 ± 0.33	0.97(0.34,1.60)			
	HL-CON	17.40 ± 3.45	20.60 ± 3.56 19.23 ± 0.34c	3.20(2.57,3.83)			
Bench Press(kg)	HL-BFR	47.67 ± 4.32	52.47 ± 5.15 59.57 ± 0.40a	4.80(4.13,5.47)	0.633	<0.001	/
	ML-BFR	55.93 ± 4.56	61.73 ± 4.89 59.94 ± 0.33b	5.80(5.13,6.47)			
	LL-BFR	56.00 ± 5.92	58.00 ± 6.48 56.14 ± 0.33	2.00(1.33,2.67)			
	HL-CON	57.47 ± 3.91	63.33 ± 4.20 59.89 ± 0.34c	5.87(5.20,6.54)			
Deadlift(kg)	HL-BFR	66.13 ± 4.87	71.27 ± 4.57 80.54 ± 0.57	5.13(4.35,5.92)	0.089	0.158	/
	ML-BFR	74.07 ± 4.38	78.87 ± 4.5 80.62 ± 0.40	4.80(4.02,5.58)			
	LL-BFR	87.00 ± 5.92	89.87 ± 6.06 79.36 ± 0.61	2.87(2.08,3.65)			
	HL-CON	76.47 ± 3.91	81.40 ± 3.85 80.88 ± 0.39c	4.93(4.15,5.72)			
Squat(kg)	HL-BFR	58.13 ± 4.87	63.00 ± 4.57 69.836 ± 0.47	4.87(4.11,5.62)	0.243	0.001	/
	ML-BFR	66.07 ± 4.38	70.93 ± 4.43 70.32 ± 0.38	4.87(4.11,5.62)			
	LL-BFR	69.00 ± 5.92	71.87 ± 6.06 68.50 ± 0.40	2.87(2.11,3.62)			
	HL-CON	68.47 ± 3.91	73.40 ± 3.85 70.54 ± 0.39c	4.93(4.18,5.69)			

Values are presented as mean ± SD. HL-BFR, 70%1RM with BFR; ML-BFR, 50%1RM with BFR; LL-BFR, 30%1RM with BFR; HL-CON, 70%1RM without BFR. ^a^indicates a significant between-group difference at post-intervention between the HL-BFR and LL-BFR groups (p < 0.05). ^b^indicates a significant between-group difference at post-intervention between the ML-BFR and LL-BFR groups (p < 0.05). ^c^indicates a significant between-group difference at post-intervention between the HL-CON and LL-BFR groups (p < 0.05).

### Explosiveness and strength

A significant main effect of time was observed for CMJ height (p < 0.001), with all groups demonstrating significant improvements following the intervention. No significant between-group differences were identified for CMJ performance.

For maximal strength outcomes, analysis of covariance (ANCOVA) revealed significant group effects for bicep curl, bench press, and squat strength (all p < 0.001), whereas no significant group effect was observed for deadlift strength. *Post hoc* comparisons indicated that the HL-BFR, ML-BFR, and HL-CON groups demonstrated significantly higher adjusted post-intervention values for bicep curl and bench press strength than the LL-BFR group (all p < 0.05), with no consistent differences among the three higher-load conditions. For squat strength, only the HL-CON group showed significantly higher adjusted post-training values compared with the LL-BFR group (p < 0.05). Specific data are presented in **Table 4**.

### RPE and heart rate

**Figure 4:**
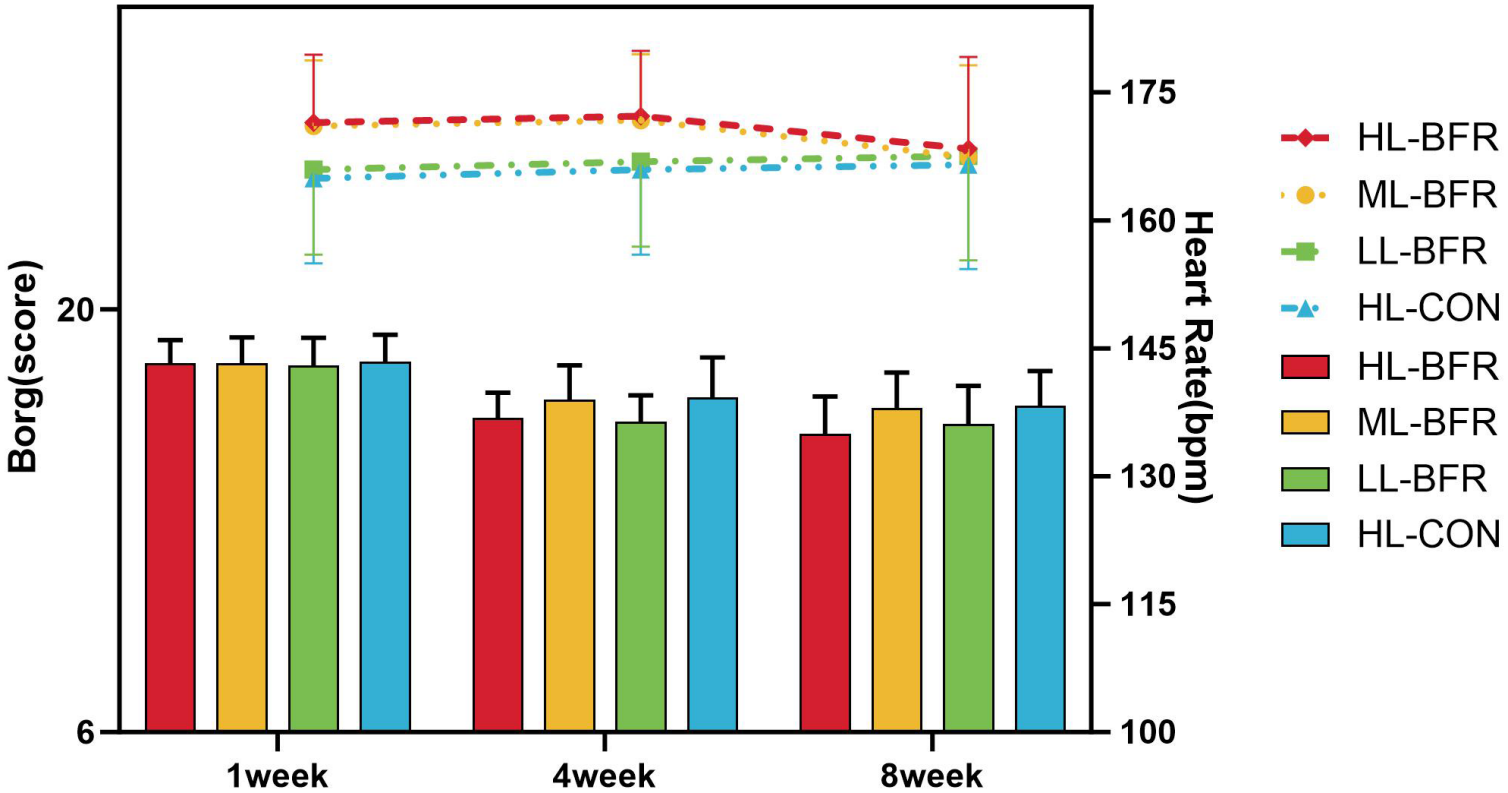
Heart rate and Borg scale during sessions. HL-BFR, 70%1RM with BFR; ML-BFR, 50%1RM with BFR; LL-BFR, 30%1RM with BFR; HL-CON, 70%1RM without BFR.

RPE and heart rate data are shown in **Figure 4**. GEE analysis showed no significant group × time interaction for RPE (Wald χ^2^=7.908, *p* = 0.245). RPE decreased significantly over time within each group (all *p* < 0.001), with no between-group differences at any time point. For heart rate, a significant group × time interaction was detected (Wald χ^2^=49.662, *p* < 0.001). However, no between-group differences were observed at individual time points. Within-group analyses indicated.

a progressive decrease in heart rate over time.

## Discussion

This study provides a comparative analysis of resistance training programs combining different load intensities with BFR in male tactical personnel. The findings suggest that ML-BFR protocols resulted in greater performance improvements than LL-BFR under the specific training structure employed. Notably, medium- and high-load BFR produced performance improvements that were generally comparable to those observed in traditional high-load resistance training, while both approaches tended to outperform low-load BFR under the present training conditions. These findings suggest that the combination of mechanical tension and BFR-induced metabolic stress may contribute to favorable neuromuscular adaptations, supporting medium-to-high load BFR as a viable strategy to optimize physical performance in tactical populations.

### The influence and potential mechanisms of different load BFR training on athletic performance

Previous research indicates that upper limb pulling strength, lower limb explosive power, and short-distance acceleration ability are important determinants of military task performance (National Strength and Conditioning Association, 2017). Prior research (Cintineo et al., 2024) has shown that while Low BFR can enhance overall military athletic performance and body composition, it remains inferior to HL-RT for improving specific strength parameters. A key finding of this study is that ML−BFR and HL−BFR training significantly improved multiple tactical−related athletic performance indicators compared to the LL−BFR group and the HL−CON group. This advantage is not only reflected in the athletic performance test results, but also shows a consistent trend with the magnitude of strength increase, training stimulus characteristics, and changes in internal load. Because resistance training performed at different relative loads elicits distinct neuromuscular adaptations (Lin et al., 2024), the superior improvements in upper-limb functional performance observed in the High and Mid groups likely reflect integrated enhancements in strength reserve and neuromuscular efficiency. Upper-limb tasks such as pull-ups and medicine ball throw collectively depend on pulling and pushing strength, trunk stability, and coordinated proximal-to-distal force transmission. Pull-ups ability, a relative strength task influenced by body mass and maximal pulling capacity (Sanchez-Moreno et al., 2016), together with medicine ball throw performance, which reflects whole-body power expression and upper-body explosive force production (Shinkle et al., 2012), improved in parallel with the larger strength adaptations observed in the HL-BFR and ML-BFR groups. Notably, the markedly greater bicep curls effect sizes (ML-BFR ηp^2^ = 0.593; HL-BFR ηp^2^ = 0.673 vs LL-BFR ηp^2^ = 0.145) suggest an expanded strength reserve that may reduce relative effort during repeated pulling actions while simultaneously supporting force transmission during ballistic pushing tasks. Collectively, these findings indicate that medium-to-high load BFR may reflect combined adaptations in strength capacity and task-specific force production, thereby enhancing tactical upper-limb functional performance. Lower-limb explosive tasks demonstrated a comparable response profile. Medium- and high-load BFR appear to confer advantages for explosive performance development, consistent with evidence linking maximal strength capacity with jump and sprint ability through increased force-generating capacity and rate of force development (Stone et al., 2003; Haff and Nimphius, 2012; Oliveira et al., 2013). The greater squat and deadlift adaptations in these groups therefore likely provided a structural and neural foundation for improved stretch–shortening cycle utilization and short-distance acceleration, aligning with prior observations that maximal strength gains underpin explosive performance enhancement (Kubo et al., 2006; Vechin et al., 2015). In contrast, the smaller improvements observed in the LL-BFR group likely reflect insufficient training stimulus rather than the ineffectiveness of low-load BFR itself. The relatively limited adaptations may therefore be related not only to reduced mechanical tension, but also to the possibility that the fixed-volume protocol (4 × 8 at 30% 1RM) did not provide sufficient fatigue accumulation or proximity to failure, both of which are considered important mediators of low-load BFR adaptations. These findings imply that comparable adaptations with low-load BFR relative to high-load conditions may require either increased training volume or training performed closer to volitional fatigue, highlighting the importance of adequate stimulus alongside mechanical tension and underscoring the critical role of sufficient mechanical tension in improving tactical performance, consistent with previous research (Harman et al., 1997; Abe et al., 2005). In addition, previous studies on post-activation potentiation and post-activation performance enhancement have suggested that resistance exercise combined with BFR may acutely enhance neuromuscular performance through increased motor unit recruitment and heightened neural activation (Zhang et al., 2023). Although the present study did not assess acute potentiation responses, such mechanisms may partly contribute to the improvements observed in explosive and sprint-related performance following repeated BFR training. However, this interpretation remains speculative and requires direct mechanistic investigation.

Combined RPE and heart rate responses indicated that BFR training was associated with a higher cardiovascular demand during the early phase of intervention, with previous studies reporting average heart rates of ~86% HRmax under BFR compared with ~83% under non-BFR conditions (Takano et al., 2005; Renzi et al., 2010). However, both subjective and objective load indicators declined over time, suggesting progressive physiological adaptation and improved cardiovascular efficiency. Long-term resistance training has been shown to induce chronic cardiovascular adjustments, while reductions in RPE may reflect enhanced neuromuscular efficiency and training tolerance (Tanimoto et al., 2005; Madarame et al., 2025). Notably, despite greater mechanical loading, the ML-BFR and HL-BFR groups did not exhibit sustained heart rate elevation or abnormal increases in RPE, indicating that this training approach can provide substantial stimulus without imposing excessive overall physiological burden.

Interestingly, adding BFR to high-load resistance training did not produce consistently greater improvements than traditional high-load training alone (Hammert et al., 2023). A possible explanation is that training at 70% 1RM already provides sufficient mechanical and neuromuscular stimulus, thereby limiting the additional contribution of BFR-induced metabolic stress. In addition, the trained status of the participants and the relatively short intervention duration may have reduced the likelihood of detecting between-condition differences.

To explore potential mechanisms underlying training adaptations, resting levels of GH, cortisol, and testosterone were assessed. No significant chronic changes were observed in any group, consistent with previous resistance and BFR training studies (Alvarez and de Paz, 2017). However, the present study only evaluated resting hormonal concentrations before and after the intervention period and did not assess acute post-exercise hormonal responses. Therefore, the possibility that BFR influenced transient endocrine responses during or immediately following exercise cannot be excluded. Previous evidence has suggested that resistance training adaptations may depend more on repeated acute hormonal fluctuations and local intramuscular signaling than on sustained elevations in resting hormone levels (Kraemer and Ratamess, 2005). In addition, circulating hormone concentrations may be influenced by several confounding factors, including nutritional status, sleep, and testing time. Although testing procedures were standardized as much as possible, these factors may still have contributed to variability in hormonal measurements. Collectively, the stable resting hormonal profile observed in this study suggests that the observed adaptations were more likely related to neuromuscular and local muscular mechanisms rather than chronic systemic endocrine alterations.

### Limitations

Despite the rigorous design of this study, several limitations should be acknowledged. First, the cohort consisted exclusively of male tactical personnel recruited under strict inclusion criteria, which enhanced occupational relevance but limited the generalizability of the findings to female populations and other training backgrounds. This limitation is particularly relevant given reported sex-related differences in responses to resistance training with and without BFR, including adaptations in strength, body composition, and hormonal profiles (Bishop et al., 1987; Fernandez-Gonzalo et al., 2014; Walker et al., 2017). In addition, the relatively small sample size and short intervention duration may have reduced the ability to detect subtle between-group differences.

Second, only a 70% 1RM non-BFR control condition was included. Therefore, the independent effects of load intensity and BFR application could not be fully separated across all loading conditions.

Third, only resting hormonal concentrations were assessed before and after the intervention, whereas acute post-exercise endocrine responses were not evaluated. In addition, body composition was assessed using bioelectrical impedance analysis, and the small segmental lean mass changes observed should therefore be interpreted cautiously given the inherent measurement variability of BIA-based methods, despite standardized testing procedures.

## Conclusions

This study demonstrated that, under a fixed training structure, resistance training combined with BFR at 50% and 70% 1RM produced greater improvements in performance outcomes than 30% 1RM with BFR in male tactical personnel. However, no consistent additional benefit was observed when BFR was added to high-load resistance training. These findings should be interpreted as comparisons among specific load–BFR combinations rather than evidence of the isolated effect of BFR. Within the constraints of the present protocol, medium-load BFR may represent a practical strategy for balancing training stimulus and mechanical load. Further studies using fully controlled designs are required to clarify the independent effects of BFR and to optimize training prescriptions.

## Data Availability

The original contributions presented in the study are included in the article/supplementary material. Further inquiries can be directed to the corresponding authors.

## References

[B1] Abe T. Kawamoto K. Yasuda T. Kearns C. F. Midorikawa T. Sato Y. (2005). Eight days KAATSU-resistance training improved sprint but not jump performance in collegiate male track and field athletes. Int. J. KAATSU Train Res. 1, 19–23. doi: 10.3806/ijktr.1.19

[B2] Alvarez L. de Paz J. A. (2017). Muscle damage responses and adaptations to eccentric-overload resistance training. BMC Sports Sci. Med. Rehabil 9, 14. doi: 10.1007/s00421-014-2836-7 28785411

[B3] Bishop P. Cureton K. Collins M. (1987). Sex difference in muscular strength in equally-trained men and women. Ergonomics 30, 675–687. doi: 10.1080/00140138708969760 3608972

[B4] Cintineo H. P. Chandler A. J. Mastrofini G. F. Lints B. S. McFadden B. A. Arent S. M. (2024). Effects of minimal-equipment resistance training and blood flow restriction on military-relevant performance outcomes. J. Strength Cond Res. 38, 55–65. doi: 10.1519/jsc.0000000000004596 38085621

[B5] Cook C. J. Kilduff L. P. Beaven C. M. (2014). Improving strength and power in trained athletes with 3 weeks of occlusion training. Int. J. Sports Physiol. Perform. 9, 166–172. doi: 10.1123/ijspp.2013-0018 23628627

[B6] Fernandez-Gonzalo R. Lundberg T. R. Alvarez-Alvarez L. de Paz J. A. (2014). Muscle damage responses and adaptations to eccentric-overload resistance exercise in men and women. Eur. J. Appl. Physiol. 114, 1075–1084. doi: 10.1007/s00421-014-2836-7 24519446

[B7] Grgic J. Lazinica B. Schoenfeld B. J. Pedisic Z. (2020). Test–retest reliability of the one-repetition maximum (1RM) strength assessment: a systematic review. Sports Med. - Open 6, 31. doi: 10.1186/s40798-020-00260-z 32681399 PMC7367986

[B8] Haff G. G. Nimphius S. (2012). Training principles for power. Strength Cond J. 34, 2–12. doi: 10.1519/ssc.0b013e31826db467 38604988

[B9] Hammert W. B. Moreno E. N. Martin C. C. Jessee M. B. Buckner S. L. (2023). Skeletal muscle adaptations to high-load resistance training with pre-exercise blood flow restriction. J. Strength Cond Res. 37, 2381–2388. doi: 10.1519/jsc.0000000000004553 37535935

[B10] Harman E. A. Frykman P. N. Palmer C. Lammi E. Reynolds K. L. (1997). Effects of a specifically designed physical conditioning program on the load carriage and lifting performance of female soldiers. Mil Med. 162, 423–430. doi: 10.1249/00005768-199205001-00774

[B11] Harman E. A. Gutekunst D. J. Frykman P. N. Nindl B. C. Alemany J. A. Mello R. P. . (2008). Effects of two different eight-week training programs on military physical performance. J. Strength Cond Res. 22, 524–534. doi: 10.1519/jsc.0b013e31816347b6 18550970

[B12] Hopkins W. G. Marshall S. W. Batterham A. M. Hanin J. (2009). Progressive statistics for studies in sports medicine and exercise science. Med. Sci. Sports Exercise 41, 3–12. doi: 10.1249/mss.0b013e31818cb278 19092709

[B13] Karabulut M. Bemben D. A. Sherk V. D. Anderson M. A. Abe T. Bemben M. G. (2011). Effects of high-intensity resistance training and low-intensity resistance training with vascular restriction on bone markers in older men. Eur. J. Appl. Physiol. 111, 1659–1667. doi: 10.1007/s00421-010-1796-9 21207053

[B14] Knapik J. J. (1997). The influence of physical fitness training on the manual material handling capability of women. Appl. Ergon 28, 339–345. doi: 10.1016/s0003-6870(97)00004-5 9414374

[B15] Kraemer W. J. Ratamess N. A. (2005). Hormonal responses and adaptations to resistance exercise and training. Sports Med. 35, 339–361. doi: 10.2165/00007256-200535040-00004 15831061

[B16] Kubo K. Komuro T. Ishiguro N. Tsunoda N. Sato Y. Ishii N. . (2006). Effects of low-load resistance training with vascular occlusion on the mechanical properties of muscle and tendon. J. Appl. Biomech 22, 112–119. doi: 10.1123/jab.22.2.112 16871002

[B17] Laurentino G. Ugrinowitsch C. Aihara A. Fernandes A. Parcell A. Ricard M. . (2008). Effects of strength training and vascular occlusion. Int. J. Sports Med. 29, 664–667. doi: 10.1055/s-2007-989405 18213536

[B18] Laurentino G. C. Ugrinowitsch C. Roschel H. Aoki M. S. Soares A. G. Neves M. . (2012). Strength training with blood flow restriction diminishes myostatin gene expression. Med. Sci. Sports Exerc 44, 406–412. doi: 10.1249/mss.0b013e318233b4bc 21900845

[B19] Lin Y. T. Wong C. M. Chen Y. C. Chen Y. Hwang I. S. (2024). Differential training benefits and motor unit remodeling in wrist force precision tasks following high and low load blood flow restriction exercises under volume-matched conditions. J. NeuroEng Rehabil 21, 123. doi: 10.1186/s12984-024-01419-5 39030574 PMC11264616

[B20] Loenneke J. P. Pujol T. J. (2009). The use of occlusion training to produce muscle hypertrophy. Strength Cond J. 31, 77–84. doi: 10.1519/ssc.0b013e3181a5a352 38604988

[B21] Madarame E. Reboursière E. Gauthier A. Hodzic A. (2025). Association between muscle strength gains and biventricular cardiac remodeling in response to high-intensity resistance training in healthy untrained males: a longitudinal study. BMC Sports Sci. Med. Rehabil 17, 116. doi: 10.1186/s13102-025-01165-8 40336139 PMC12057098

[B22] Martín-Hernández J. Marín P. J. Menéndez H. Ferrero C. Loenneke J. P. Herrero A. J. (2013). Muscular adaptations after two different volumes of blood flow-restricted training. Scand. J. Med. Sci. Sports 23, e114–e120. 23278841 10.1111/sms.12036

[B23] Mattocks K. T. Jessee M. B. Counts B. R. Buckner S. L. Mouser J. G. Dankel S. J. . (2017). The effects of upper body exercise across different levels of blood flow restriction on arterial occlusion pressure and perceptual responses. Physiol. Behav. 171, 181–186. doi: 10.1016/j.physbeh.2017.01.015 28088558

[B24] National Strength and Conditioning Association (2017). NSCA’s essentials of tactical strength and conditioning (Champaign (IL: Human Kinetics).

[B25] Ohta H. Kurosawa H. Ikeda H. Iwase Y. Satou N. Nakamura S. (2003). Low-load resistance muscular training with moderate restriction of blood flow after anterior cruciate ligament reconstruction. Acta Orthop. Scand. 74, 62–68. doi: 10.1080/00016470310013680 12635796

[B26] Oliveira F. B. Oliveira A. S. Rizatto G. F. Denadai B. S. (2013). Resistance training for explosive and maximal strength: effects on early and late rate of force development. J. Sports Sci. Med. 12, 402–408. 24149144 PMC3772581

[B27] Patterson S. D. Hughes L. Warmington S. Burr J. Scott B. R. Owens J. . (2019). Blood flow restriction exercise: considerations of methodology, application, and safety. Front. Physiol. 10, 533. doi: 10.3389/fphys.2019.00533 31156448 PMC6530612

[B28] Pişkin N. E. Yavuz G. Aktuğ Z. B. Aldhahi M. I. Al-Mhanna S. B. Gülü M. (2024). The effect of combining blood flow restriction with the Nordic hamstring exercise on hamstring strength: randomized controlled trial. J. Clin. Med. 13, 2035. 38610800 10.3390/jcm13072035 PMC11012977

[B29] Reeves G. V. Kraemer R. R. Hollander D. B. Clavier J. Thomas C. Francois M. . (2006). Comparison of hormone responses following light resistance exercise with partial vascular occlusion and moderately difficult resistance exercise without occlusion. J. Appl. Physiol. 101, 1616–1622. doi: 10.1152/japplphysiol.00440.2006 16902061

[B30] Renzi C. P. Tanaka H. Sugawara J. (2010). Effects of leg blood flow restriction during walking on cardiovascular function. Med. Sci. Sports Exerc 42, 726–732. doi: 10.1249/mss.0b013e3181bdb454 19952840 PMC2888901

[B31] Sanchez-Moreno M. Pareja-Blanco F. Diaz-Cueli D. González-Badillo J. J. (2016). Determinant factors of pull-up performance in trained athletes. J. Sports Med. Phys. Fitness 56, 825–833. 26176615

[B32] Shinkle J. Nesser T. W. Demchak T. J. McMannus D. M. (2012). Effect of core strength on the measure of power in the extremities. J. Strength Cond Res. 26, 373–380. doi: 10.1519/jsc.0b013e31822600e5 22228111

[B33] Stone M. H. O’Bryant H. S. McCoy L. Coglianese R. Lehmkuhl M. Schilling B. (2003). Power and maximum strength relationships during performance of dynamic and static weighted jumps. J. Strength Cond Res. 17, 140–147. doi: 10.1519/00124278-200302000-00022 12580669

[B34] Takano H. Morita T. Iida H. Asada K. I. Kato M. Uno K. . (2005). Hemodynamic and hormonal responses to a short-term low-intensity resistance exercise with the reduction of muscle blood flow. Eur. J. Appl. Physiol. 95, 65–73. doi: 10.1007/s00421-005-1389-1 15959798

[B35] Takarada Y. Tsuruta T. Ishii N. (2004). Cooperative effects of exercise and occlusive stimuli on muscular function in low-intensity resistance exercise with moderate vascular occlusion. Jpn. J. Physiol. 54, 585–592. doi: 10.2170/jjphysiol.54.585 15760491

[B36] Tanimoto M. Madarame H. Ishii N. (2005). Muscle oxygenation and plasma growth hormone concentration during and after resistance exercise: comparison between KAATSU and other types of regimen. Int. J. KAATSU Train Res. 1, 51–56. doi: 10.3806/ijktr.1.51

[B37] Vechin F. C. Libardi C. A. Conceição M. S. Damas F. R. Lixandrão M. E. Berton R. P. B. . (2015). Comparisons between low-intensity resistance training with blood flow restriction and high-intensity resistance training on quadriceps muscle mass and strength in elderly. J. Strength Cond Res. 29, 1071–1076. doi: 10.1519/jsc.0000000000000703 25264670

[B38] Walker A. J. McFadden B. A. Sanders D. J. Hofacker M. L. Bello M. L. Poyssick A. N. . (2017). Workload, energy expenditure, and biomarker differences in Division I male and female soccer players. Med. Sci. Sports Exerc 49, 571. doi: 10.1249/01.mss.0000518485.92630.f1 30958151

[B39] Yasuda T. Ogasawara R. Sakamaki M. Ozaki H. Sato Y. Abe T. (2011). Combined effects of low-intensity blood flow restriction training and high-intensity resistance training on muscle strength and size. Eur. J. Appl. Physiol. 111, 2525–2533. doi: 10.1007/s00421-011-1873-8 21360203

[B40] Zhang J. Zhou R. Zhao N. Li Y. Liu H. Zhang W. . (2023). Acute effects of blood flow restriction with whole-body vibration on sprint, muscle activation and metabolic accumulation in male sprinters. Front. Physiol. 14, 1149400. doi: 10.3389/fphys.2023.1149400 37035675 PMC10074852

